# Estimating the average distribution of Antarctic krill *Euphausia superba* at the northern Antarctic Peninsula during austral summer and winter

**DOI:** 10.1007/s00300-022-03039-y

**Published:** 2022-04-15

**Authors:** V. Warwick-Evans, S. Fielding, C. S. Reiss, G. M. Watters, P. N. Trathan

**Affiliations:** 1 British Antarctic Survey, High Cross, Madingley Road, Cambridge, CB3 0ET UK; 2 Antarctic Ecosystem Research Division, Southwest Fisheries Science Center, National Marine Fisheries Service, National Oceanic and Atmospheric Administration, 8901 La Jolla Shores Drive, La Jolla, CA 92037-1508 USA

**Keywords:** Fisheries management, Habitat modelling, Predator requirements, Seasonal distribution

## Abstract

This study was performed to aid the management of the fishery for Antarctic krill *Euphausia superba*. Krill are an important component of the Antarctic marine ecosystem, providing a key food source for many marine predators. Additionally, krill are the target of the largest commercial fishery in the Southern Ocean, for which annual catches have been increasing and concentrating in recent years. The krill fishery is managed by the Commission for the Conservation of Antarctic Marine Living Resources (CCAMLR), which has endorsed a new management framework that requires information about the spatial distribution and biomass of krill. Here, we use krill density estimates from acoustic surveys and a GAMM framework to model habitat properties associated with high krill biomass during summer and winter in the northern Antarctic Peninsula region, an area important to the commercial fishery. Our models show elevated krill density associated with the shelf break, increased sea surface temperature, moderate chlorophyll-a concentration and increased salinity. During winter, our models show associations with shallow waters (< 1500 m) with low sea-ice concentration, medium sea-level anomaly and medium current speed. Our models predict temporal averages of the distribution and density of krill, which can be used to aid CCAMLR’s revised ecosystem approach to fisheries management. Our models have the potential to help in the spatial and temporal design of future acoustic surveys that would preclude the need for modelled extrapolations. We highlight that the ecosystem approach to fisheries management of krill critically depends upon such field observations at relevant spatial and temporal scales.

**Supplementary Information:**

The online version contains supplementary material available at 10.1007/s00300-022-03039-y.

## Introduction

Antarctic krill (*Euphausia superba*) are the principal prey item for many Antarctic marine predators (Trathan and Hill 2016), providing a key link between phytoplankton production and higher trophic levels (Laws 1985). Krill are abundant at the circumpolar scale and are now the target of the largest fishery in the Southern Ocean (Nicol et al. 2012), with catches in the 2019/20 fishing season exceeding 450,000 t (CCAMLR 2020). In recent years, krill fishery operations have become more concentrated within the Antarctic Peninsula region, an important feeding and spawning ground for krill (Atkinson et al. 2008; Perry et al. 2019; Siegel 1988), and for numerous krill predators (Trathan and Hill 2016).

Krill biomass varies greatly (≫ 1 order of magnitude), at both local (survey scale, e.g. Reiss et al. 2008) and regional (ocean basin scale, e.g. Hewitt et al. 2004) scales (Atkinson et al. 2008; Ross et al. 2014; Reiss et al. 2017). Moreover, there is conflicting evidence of a long-term decline in abundance (Atkinson et al. 2004, but see Kinzey and Watters 2013; Cox et al. 2018; Krafft et al. 2021).

Inter-annual variability in krill biomass is marked, especially in summer (Reiss et al. 2008), which is likely to be a result of periodicity in their lifecycle dynamics. In the west Antarctic Peninsula region, krill exhibit a 5–8 year cycle in recruitment, with oscillations in biomass exceeding an order of magnitude (Hewitt et al. 2003; Ryabov et al. 2017). Modelling studies suggest that this cycle may be a result of intraspecific competition for food, others have suggested that these cycles are modulated by climatological factors including sea-ice duration (Ross et al. 2014; Ryabov et al. 2017). Climatic oscillations that may affect the duration and extent of winter sea-ice and short-term ecosystem dynamics include the El Niño Southern Oscillation (ENSO) and the Southern Annular Mode (SAM) (e.g. Trathan and Murphy 2002; Loeb et al. 2009; Loeb and Santora 2013; Saba et al. 2014). Long-term increases in the frequency of years with reduced sea-ice duration, as a result of positive trends in air and sea surface temperatures (SST), have also been observed (Vaughan et al. 2003; Meredith and King 2005; Stammerjohn et al. 2008). As such, variability in climatic events such as ENSO and SAM may explain the inter-annual variation in krill biomass (Murphy et al. 2007), whilst long-term trends in the duration of winter sea-ice may result in long-term population change (Atkinson et al. 2004).

Long-term trends in the duration of winter sea-ice at the Antarctic Peninsula have been implicated in long-term population declines in krill (Atkinson et al. 2004). Thus, links may exist between sea-ice and krill at various life-history stages, including recruitment, spawning and overwintering (Daly 1990; Kawaguchi and Satake 1994; Loeb et al. 1997; Ducklow et al. 2006). Additionally, both adult and juvenile krill may rely on sea-ice biota for food during periods when primary productivity in the water column is low (Quetin et al. 1996; Daly 2004), although Walsh et al. 2020 found that post-larval krill do not rely on sea-ice resources for overwinter survival. As such, years of reduced sea-ice may reduce krill biomass as a result of decreased krill recruitment and spawning, higher mortality of larval krill or a reduced food supply (Loeb et al. 1997; Veytia et al. 2020). As such, whilst cohorts age and are depleted by natural mortality, they may only be replaced at irregular intervals (Reid et al. 2010).

In addition to extreme inter-annual variability in krill biomass, krill may perform seasonal migrations from offshore waters in summer to on-shelf habitats, often under sea-ice or in the marginal ice zone, in winter (Marschall 1988; Siegel 1988; Lascara et al. 1999; Nicol 2006).

Understanding these seasonal differences in distribution and abundance is vital for management. The Commission for the Conservation of Antarctic Marine Living Resources (CCAMLR), established in 1981, has the express aim of managing the krill fishery in a way that minimises the impacts of harvesting on krill and its predators. To this end, CCAMLR has recently endorsed a new management framework that integrates spatial data relating to krill biomass and predator foraging to provide an ecosystem approach to management (CCAMLR 2019). One of the challenges associated with implementing this management framework is the requirement for fine-scale information regarding the distribution and abundance of krill. Although surveys to estimate krill abundance date back to the *Discovery Investigations* in the 1920s and 1930s, many of them do not adequately sample the areas used by the modern commercial fishery, areas important for krill life-history or areas important for dependent predators. One way to overcome the limitations of existing survey data, to fill in the gaps, is through modelling.

Habitat models can provide a robust approach to extrapolating species distributions into nearby areas. They involve modelling the relationship between animal density and spatio-environmental covariates and estimating density across a wider area according to available environmental conditions. Previous models conducted across a variety of spatial scales show summer krill density may be associated with depth, distance to the shelf break, current speed, sea surface temperature, salinity, eddy kinetic energy, sea-level anomaly and chlorophyll-a concentration (Trathan et al. 2003; Santora et al. 2012; Silk et al. 2016). These habitat characteristics are likely to represent both broad-scale features which drive krill distribution, and meso-scale features which concentrate krill (Santora et al. 2012).

Most models have explored habitat relationships in the summer, whilst few studies have investigated the drivers behind the winter distribution of krill. Nevertheless, the same environmental covariates may be important.

Understanding average conditions is useful for management of the krill fishery, particularly because other ecosystem components [e.g. predator populations, Trathan et al. 2018; Warwick-Evans et al. 2022)] will depend upon predictability of the available krill stock. Understanding spatial and temporal overlap in demand from both predators and the fishery is a key part of the new CCAMLR approach for management (CCAMLR 2019). We recognise that habitat models are imperfect and do not encapsulate all the information needed for management. For example, a single habitat model for krill cannot provide information about inter- or intra-annual variability. Moreover, models cannot resolve spatial details at scales less than observed data or covariate data. However, the approach endorsed by CCAMLR (CCAMLR 2019) requires information about the spatial distribution and biomass of krill presented as an average representation of krill for inclusion in the new management framework, with a model for summer and a separate model for winter.

Here, we create habitat models to associate the seasonal distribution of Antarctic krill with habitat characteristics and use these to predict the distribution of krill across the Antarctic Peninsula region, extrapolating into un-surveyed areas. We discuss how this information can aid the ecosystem approach to fisheries management in a highly variable, dynamic and sensitive ecosystem.

## Methods

### Study area and sampling approach

#### Antarctic krill density data

**Fig. 1:**
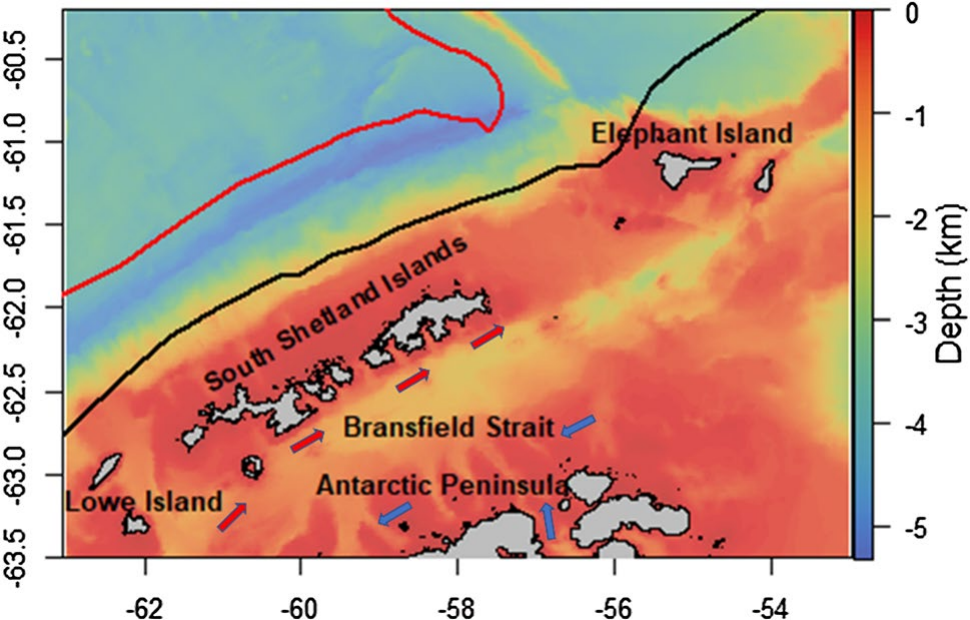
The north Antarctic Peninsula region. Including SACCF (red) SACCF southern Boundary (black), Bransfield current system indicating Weddell-influenced water (blue arrows) and Bellingshausen-influenced water (red arrows) adapted from (Sangrà et al. 2011) and (Orsi et al. 1995)

**Fig. 2:**
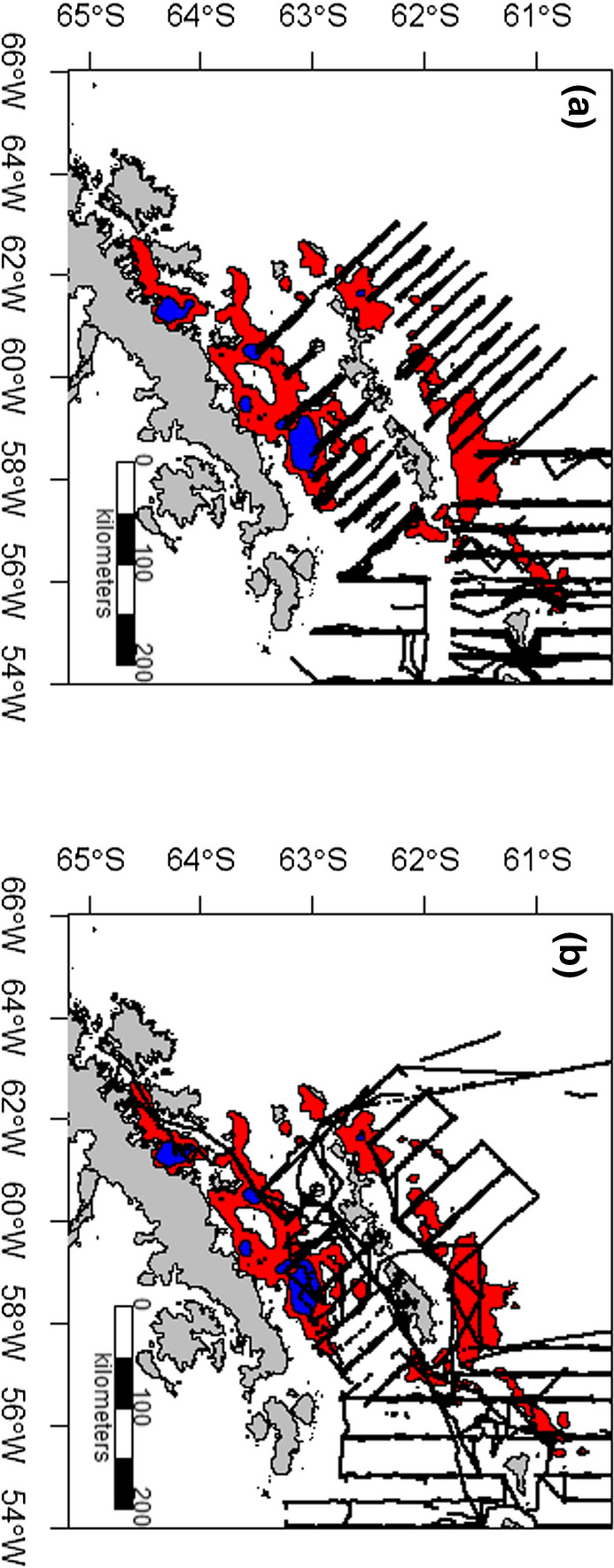
Acoustic transects sampled for Antarctic krill *Euphausia superba* by US AMLR. Transects occured during **a** summer between 1999 and 2011, **b** winter between 2012 and 2016. See (Reiss et al. 2008) for survey design, overlaid on krill fishery 95% summer usage (red) and 50% summer usage (blue) between 2010 and 2015 (after Trathan et al. 2018)

The U.S. Antarctic Marine Living Resources (AMLR) Program conducted annual ship-based monitoring surveys around the South Shetland Islands and the northern Antarctic Peninsula region (Fig. 1) during austral summer (January–March) between 1999 and 2011 and during winter (August–September) between 2012 and 2016 (Fig. 2). Acoustic transects were sampled during transits (ship speed ~ 10-knots) between a fixed grid of oceanographic and biological sampling stations. The rational for these surveys was to estimate the biomass of Antarctic krill. The survey methods, data processing and net processing details are described by Reiss et al. (2008); Cossio et al. (2011) and Reiss et al. (2017). However, briefly, zooplankton samples at each biological station were collected using a 2.5 m^2^ (505-µm mesh) Isaac-Kidd midwater trawl. Krill and other taxa (e.g. other krill species) were enumerated, whilst Antarctic krill were sexed, staged for maturity and measured to the nearest mm. Multi-frequency (200 kHz, 120 kHz and 38 kHz) acoustic data were used to identify krill and estimate biomass density (wet weight gm^−2^) with estimates integrated from 250 m to the near-surface and over 1 nautical mile horizontal elementary sampling units, using the Stochastic Wave Born Approximation approach (Cossio et al. 2011) for target strength. Local daylight was used to assign transects and net tows to daytime or nighttime. Because of seasonal variability in the vertical distribution of Antarctic krill, the 250 m integration depth (which is limited by the vertical resolution of the 200-kHz echosounder) may have underestimated the biomass density of Antarctic krill (Reiss et al. 2017; Bernard et al. 2019). However, the spatial distribution of biomass density would not have been affected greatly. This acoustic approach can resolve krill between about 20 to 65 mm in length.

#### Environmental covariates

Both static and dynamic environmental variables were used in our analyses (Table 1). Biologically meaningful contemporaneous environmental covariates previously identified to influence the distribution of krill (e.g. Trathan et al. 2003; Silk et al. 2016) were extracted for each grid cell using R packages *ncdf4* (Pierce 2019) and *raster* (Hijmans and van Etten 2014). The shelf break was classed as the 1000 m depth contour, with values on-shelf negative, and those off-shelf positive. The deep trenches (> 1000 m) within the Bransfield Strait (Fig. 1) were classed as off-shelf. Multicollinearity amongst covariates was evaluated using Variance Inflation Factors (VIFs) and concurvity was also measured. Depth and Distance to shelf break both had VIFs > 0.5 (0.61 and 0.62, respectively), and thus were not included in the same models. Given the similarity in VIFs for these variables, both were evaluated and the variable that most improved model performance was included. Concurvity estimates were all < 0.6 which suggests that none of the predictors could be approximated by the other predictors in the model.

**Table 1:** Explanatory variables evaluated in model selection

Covariate	Spatial resolution km	Temporal resolution	Source
Bathymetry (depth)	0.3	NA	http://quantarctica.npolar.no
Slope	0.3	NA	Calculated in R using bathymetry data
Distance to shelf break (1000 m, the 1000 m contour in the Bransfield Strait is classed as shelf break)	0.3	NA	Calculated in R using bathymetry data
Salinity (surface)	~ 4.3 × 9.2	Daily	www.marine.copernicus.eu
Chlorophyll-a (Chl, surface)	~ 4 × 4	Daily	www.marine.copernicus.eu
Sea surface temperature (SST)	~ 2.2 × 4.6	Daily	www.marine.copernicus.eu
Mean sea level anomaly (SLA)	~ 13 × 28	Daily	www.marine.copernicus.eu
Current speed (C)	~ 4.3 × 9.2	Daily	Calculated in R using data from www.marine.copernicus.eu
Eddy kinetic energy‡ (EKE)	~ 4.3 × 9.2	Daily	Calculated in R using data from www.marine.copernicus.eu
Sea-ice concentration*	0.3	Daily	www.marine.copernicus.eu

Evaluated for statistical models to predict the distribution and density of Antarctic krill *Euphausia superba* around the South Shetland Islands and West Antarctic Peninsula. For each of the temporally dynamic covariates (Sea surface temperature, Sea-level anomaly, Salinity, Chlorophyll-a concentration, Current speed, Eddy kinetic energy), both real-time values and 11-year summer climatologies were evaluated independently, and model selection continued using only the highest scoring of the two. During summer, the value for chlorophyll during the previous 2 months (termed chlorophyll lag 1 and chlorophyll lag 2) was also extracted to investigate any lag between chlorophyll concentration and krill density. This was not possible during winter as the majority of chlorophyll data from June and July were missing due to sea-ice cover. During winter, the sea-ice concentrations of 2 weeks, 1 month and 2 months preceding data collection were evaluated

*Winter only

^**‡**^EKE was removed from the model selection after the first round of model testing due to being highly correlated with current speed

#### Data processing and analysis

We estimate a temporal average of krill density, separately for both summer and winter, identifying which areas show higher (or lower) abundance on average. Independently for each year (and season), krill density data were binned into a 4 × 4 km orthogonal grid. The mean krill density within each grid cell was calculated for each season and each year. Gridding the acoustic data at the scale of the environmental variables avoids pseudo-replication, which would otherwise occur, given that multiple acoustic observations occur within the spatial scale of the environmental data. Gridding the data also provides a means of reducing spatial autocorrelation in model residuals. We selected the spatial resolution (4 km) as this was the approximate scale of the majority of the covariate data. We did not aggregate samples across years, given that each acoustic survey represents a unique time with a unique combination of environmental variables.

We used General Additive Mixed Models (GAMM) to model the relationship between krill density and environmental covariates using R package *mgcv* (Wood 2011), using a cubic regression spline smoothing algorithm. Krill density data were heavily skewed towards near-zero values, consequently, a Tweedie error structure was used, with a log link function and where the Tweedie parameter was estimated during model fitting. Survey year was included as a random effect in the models to account for inter-annual variation. Model selection was performed using Maximum Likelihood smoothing, and the final model was re-fit using REML for smoothness estimation. To reduce model overfitting, the number of knots was limited to between 3 and 7: For each covariate, the GAMM was run without limiting the knots, and the response curve was plotted in order to identify the shape of the relationship between the covariate and the dataset. Subsequently, the number of knots was set to between 3 and 7 in turn and evaluated visually. The selected value was that where the curve resembles the same overall pattern as shown in the unlimited data, whilst remaining biologically plausible (i.e. a single peak or a directional relationship, rather than a wiggly line). Model performance was evaluated using AIC (corrected using the algorithm developed by Wood et al. 2016) and normalised root mean square error (NMRSE). For comparison, model selection by sixfold cross validation was also evaluated, using NRMSE as the evaluation metric (see ESM 1). NRMSE represents the mean difference between predicted and observed values, standardised using the range of the latter. Lower values of NMRSE indicate a better model fit. Model selection followed a manual forwards stepwise selection approach: Each of the covariates was modelled independently and ranked according to AIC and NRMSE value, and the highest-ranking covariate was selected (i.e. with the lowest AIC and NRMSE). Each of the remaining covariates were added in turn to the best model, and the covariate was retained in the model if the AIC and NRMSE value decreased. This process continued, adding more covariates until the NRMSE value no longer decreased. The model residuals from the final model were tested for spatial autocorrelation using Auto Correlation Function (ACF) and Partial ACF (PACF) plots. The final model was used to predict the distribution and density of krill across the study area.

## Results

### Summer

**Fig. 3:**
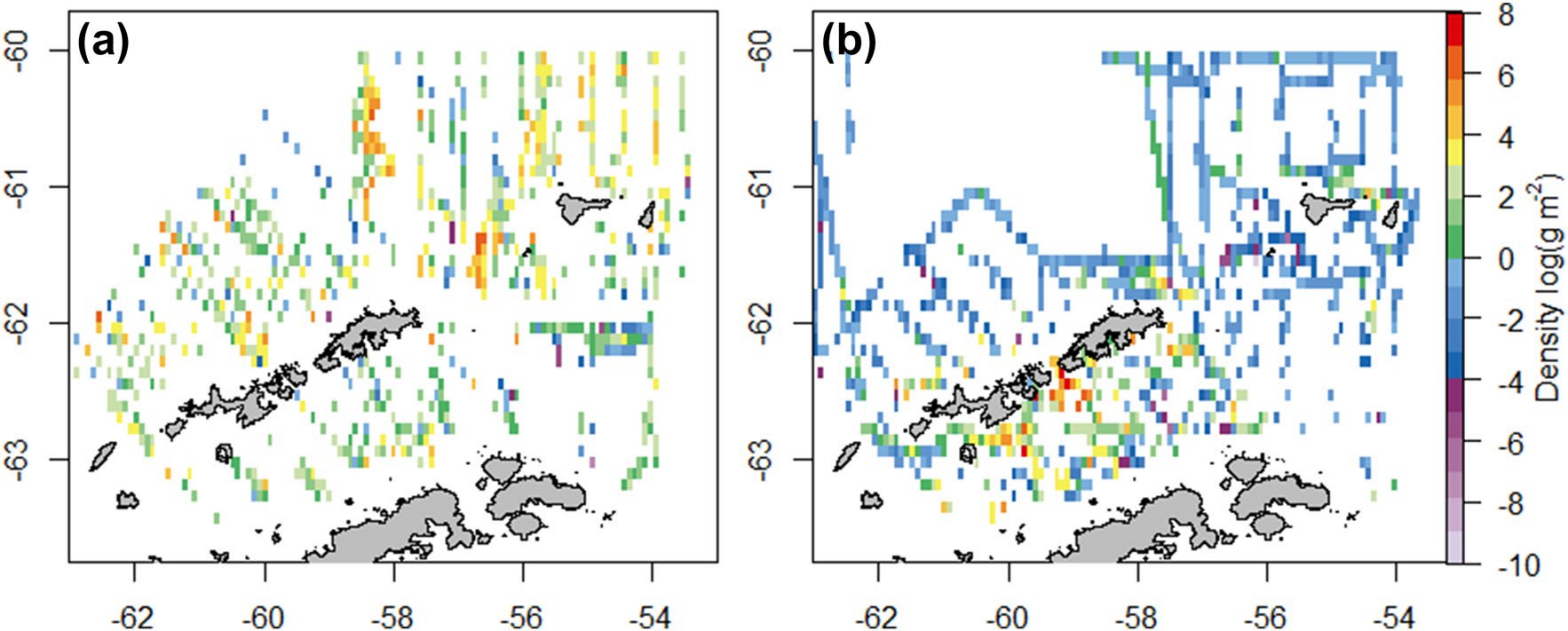
Density observations of Antarctic krill *Euphausia superba*. Mean krill density (log) averaged across all survey years during **a** summer (1999 to 2011), **b** winter (2012–2016)

An average of 1537 (± 518) grid cells were sampled each year in summer, and the observed krill density was highly variable between grid cells (Fig. 3), and between years (ESM 2). However, elevated values were consistently observed around Elephant Island and towards the southern ends of transects approaching the Peninsula and South Shetland Islands, with high inter-annual variation in maximum krill density (ESM 3).

**Fig. 4:**
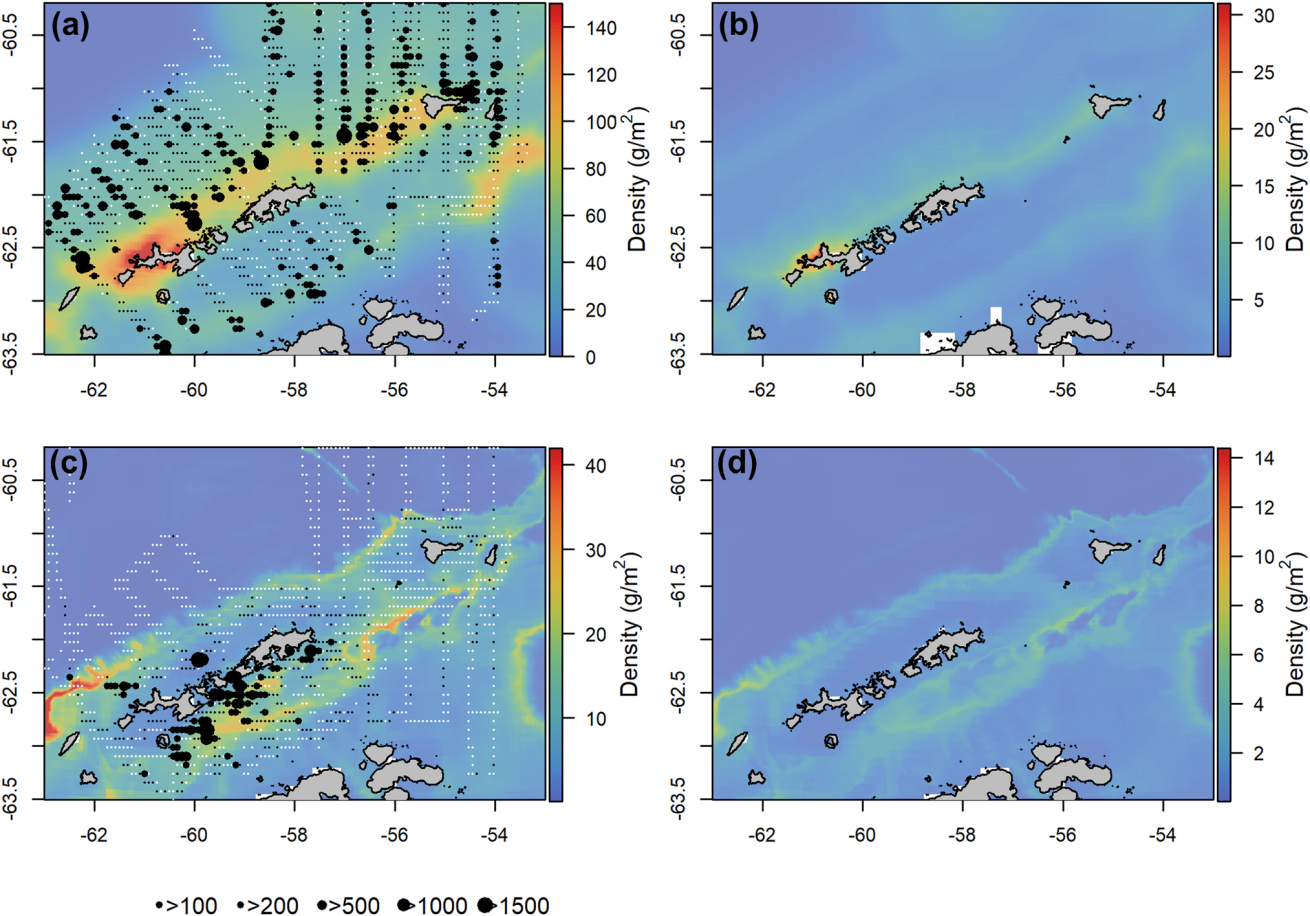
Predicted density and distribution of Antarctic krill *Euphausia superba*. Results from gamm models **a** during summer, with salinity, distance to shelf break, sea surface temperature and chlorophyll-a concentration as environmental covariates, and **b** summer standard error, **c** during winter with depth, sea-level anomaly, current speed and sea-ice concentration as the environmental covariates, **d** standard error winter. Observed values are overlaid; white dots represent all grid cells sampled, black dots increase in size with density (> 10 gm^−2^, > 200 gm^−2^, > 500 gm^−2^, > 1000 gm^−2^, > 1500 gm^−2^)

Of each of the independent environmental covariates evaluated, salinity provided the lowest AIC and NRMSE values (Table 2). The final model predicting krill density for summer included salinity, distance to shelf break, sea surface temperature, and chlorophyll-a concentration (Table 2). Increased krill density was predicted in areas with increased salinity, in shelf waters near the shelf break, with higher SSTs and moderate chlorophyll-a concentrations (ESM 4). When cross validation was used for model selection, salinity again provided the lowest NRMSE value, and forward model selection resulted in a final model with chlorophyll-a concentration, distance to shelf break and sea surface temperature (ESM 1). As such the same covariates were included in the final model, although model selection proceeded in a slightly different order. There was low spatial autocorrelation in the model residuals (ESM 5). Predictions from the model highlight the shelf break, Elephant Island and nearshore waters along the Antarctic Peninsula and South Shetland Islands as areas of elevated krill density (Fig. 4). The predicted summer density was lowest in off-shelf waters in the Drake passage, north of 61.5 °S, in the deep, central Bransfield Strait and towards the tip of the Antarctic Peninsula. Spatial error was highest in areas with the highest predicted krill density (Fig. 4).

**Table 2:** Model selection evaluations

Covariate	Summer		Winter	
	AIC	NRMSE	AIC	NRMSE
**S(salinity, *k* = 5)**	**46,251**	**0.0479**	11,270	0.0653
S(distance to shelf break, *k* = 7)	46,318	0.0482	11,429	0.0651
**S(depth, *k* = 4/5)**	46,377	0.0482	**11,216**	**0.0650**
S(sea surface temperature, *k* = 4)	46,412	0.0483	11,530	0.0653
S(chlorophyll, *k* = 5)	46,425	0.0483	11,742	0.0654
S(mean sea level anomaly, *k* = 4/6)	46,428	0.0483	11,332	0.0655
S(slope, *k* = 4)	46,435	0.0483	11,915	0.0906
S(current speed, *k* = 4)	46,437	0.0483	11,873	0.0655
S(Eddy kinetic energy, *k* = 4)	46,438	0.0484	11,896	0.0658
S(chlorophyll lag 1, *k* = 5)	46,438	0.0484	NA	NA
S(chlorophyll lag 2, *k* = 4)	46,441	0.0490	NA	NA
S(sea-ice concentration, *k* = 4)	NA	NA	11,727	0.0655
S(sea-ice concentration lag 2 week, *k* = 4)	NA	NA	11,731	0.0657
S(sea-ice concentration lag 1 month, *k* = 4)	NA	NA	11,733	0.0657
S(sea-ice concentration lag 2 months, *k* = 4)	NA	NA	11,745	0.0657
Null	46,454	0.0491	11,951	0.066
S(**salinity, *k* = 5) + S**(**distance to shelf break, *k* = 7)**	**46,072**	**0.0477**	NA	NA
S(salinity, *k* = 5) + S(depth, *k* = 4)	46,151	0.0477	NA	NA
S(salinity, *k* = 5) + S(chlorophyll, *k* = 5)	46,156	0.0477	NA	NA
S(salinity, *k* = 5) + S(sea surface temperature, *k* = 4)	46,194	0.0478	NA	NA
S(salinity, *k* = 5) + S(mean sea level anomaly, *k* = 4)	46,224	0.0478	NA	NA
S(salinity, *k* = 5) + S(current speed, *k* = 4)	46,232	0.0478	NA	NA
S(salinity, *k* = 5) + S(Slope, *k* = 4)	46,234	0.0478	NA	NA
**S(salinity, *k* = 5) + S(distance to shelf break, *k* = 7) + S(sea surface temperature, *k* = 4)**	**45,977**	**0.0476**	NA	NA
S(salinity, *k* = 5) + S(Distance to shelf break, *k* = 7) + S(chlorophyll, *k* = 5)	46,000	0.0476	NA	NA
S(salinity, *k* = 5) + S(distance to shelf break, *k* = 7) + S(Slope, *k* = 4)	46,018	0.0476	NA	NA
S(salinity, *k* = 5) + S(distance to shelf break, *k* = 7) + S(current speed, *k* = 4)	46,038	0.0476	NA	NA
S(salinity, *k* = 5) + S(distance to shelf break, *k* = 7) + S(depth, *k* = 4)	46,046	0.0477	NA	NA
S(salinity, *k* = 5) + S(distance to shelf break, *k* = 7) + S(mean sea level anomaly, *k* = 4)	46,052	0.0477	NA	NA
**S(salinity, *k* = 5) + S(distance to shelf break, *k* = 7) + S(sea surface temperature, *k* = 4) + S(chlorophyll, *k* = 5)**	**45,929**	**0.0474**	NA	NA
S(salinity, *k* = 5) + S(distance to shelf break, *k* = 7) + S(sea surface temperature, *k* = 4) + S(current speed, *k* = 4)	45,941	0.0476	NA	NA
S(salinity, *k* = 5) + S(distance to shelf break, *k* = 7) + S(sea surface temperature, *k* = 4) + S(depth, *k* = 4)	45,952	0.0476	NA	NA
S(salinity, *k* = 5) + S(distance to shelf break, *k* = 7) + S(sea surface temperature, *k* = 4) + S(slope, *k* = 4)	45,954	0.0476	NA	NA
S(salinity, *k* = 5) + S(distance to shelf break, *k* = 7) + S(sea surface temperature, *k* = 4) + S(mean sea level anomaly, *k* = 4)	45,955	0.0476	NA	NA
**S(depth, *k* = 5) + S**(**mean sea level anomaly, *k* = 6)**	**NA**	**NA**	**10,916**	**0.0631**
S(depth, *k* = 5) + S(sea-ice concentration, *k* = 4)	NA	NA	10,980	0.0637
S(depth, *k* = 5) + S(salinity, *k* = 5)	NA	NA	11,090	0.0642
S(depth, *k* = 5) + S(current speed, *k* = 4)	NA	NA	11,112	0.0649
S(depth, *k* = 5) + S(sea surface temperature, *k* = 4)	NA	NA	11,123	0.0641
S(depth, *k* = 5) + S(distance to shelf break, *k* = 7)	NA	NA	11,152	0.0649
S(depth, *k* = 5) + S(chlorophyll, *k* = 5)	NA	NA	11,182	0.0650
S(depth, *k* = 5) + S(slope, *k* = 4)	NA	NA	11,219	0.0650
**S(depth, *k* = 5) + S**(**mean sea level anomaly, *k* = 6) + S**(**Sea-ice concentration, *k* = 4)**	**NA**	**NA**	**10,837**	**0.0626**
S(depth, *k* = 5) + S(mean sea level anomaly, *k* = 6) + S(Salinity, *k* = 5)	NA	NA	10,872	0.0629
S(depth, *k* = 5) + S(mean sea level anomaly, *k* = 6) + S(sea surface temperature, *k* = 4)	NA	NA	10,883	0.0630
S(depth, *k* = 5) + S(mean sea level anomaly, *k* = 6) + S(current speed, *k* = 4)	NA	NA	10,893	0.0630
S(depth, *k* = 5) + S(mean sea level anomaly, *k* = 6) + S(distance to shelf break, *k* = 7)	NA	NA	10,893	0.0631
S(depth, *k* = 5) + S(mean sea level anomaly, *k* = 6) + S(chlorophyll, *k* = 5)	NA	NA	10,907	0.0632
S(depth, *k* = 5) + S(mean sea level anomaly, *k* = 6) + S(slope, *k* = 4)	NA	NA	10,907	0.0632
**S(depth, *k* = 5) + S**(**mean sea level anomaly, *k* = 6) + S**(**sea-ice concentration, *k* = 4) + S**(**current speed, *k* = 4)**	**NA**	**NA**	**10,762**	**0.0623**
S(depth, *k* = 5) + S(mean sea level anomaly, *k* = 6) + S(sea-ice concentration, *k* = 4) + S(chlorophyll, *k* = 5)	NA	NA	10,792	0.0623
S(depth, *k* = 5) + S(mean sea level anomaly, *k* = 6) + S(sea-ice concentration, *k* = 4) + S(salinity, *k* = 5)	NA	NA	10,795	0.0624
S(depth, *k* = 5) + S(mean sea level anomaly, *k* = 6) + S(sea-ice concentration, *k* = 4) + S(sea surface temperature, *k* = 4)	NA	NA	10,801	0.0625
S(depth, *k* = 5) + S(mean sea level anomaly, *k* = 6) + S(Sea-ice concentration, *k* = 4) + S(distance to shelf break, *k* = 7)	NA	NA	10,819	0.0626
S(Depth, *k* = 5) + S(mean sea level anomaly, *k* = 6) + S(sea-ice concentration, *k* = 4) + S(slope, *k* = 4)	NA	NA	10,838	0.0627

These determine the best environmental covariates with which to predict the density and distribution of Antarctic krill *Euphausia superba* around the South Shetland Islands and West Antarctic Peninsula. AIC and Normalised Root Mean Square Error (NRMSE) were used to evaluate models. The results show the forward model selection process, and the best performing models for each number of covariates are highlighted in bold. The number of knots in the smoothness parameter is given, and where summer and winter differ the summer value is the first of the two given, and the winter value the second

### Winter

An average of 560 (± 237) grid cells were sampled each year in winter, and the observed krill density was highly variable between grid cells (Fig. 3), and between years (ESM 2). However, elevated values were consistently observed around the South Shetland Islands and in the Bransfield Strait, with high inter-annual variation in maximum krill density (ESM 3).

Of each of the independent environmental covariates, depth provided the lowest AIC and NRMSE values. The final model for winter included depth, sea-level anomaly, sea-ice concentration and current speed (Table 2). Increased krill density was associated with shallow water (< 1500 m) with moderate sea level anomaly, low sea-ice concentration and moderate current speed (ESM 4). When cross validation was used for model selection, depth again provided the lowest NRMSE value, and the final model included sea-level anomaly, sea-ice concentration and chlorophyll-a (ESM 1). As such, the first three covariates were the same using either method of evaluation, although chlorophyll-a concentration was selected instead of current speed for the final covariate in the model. There was low spatial autocorrelation in the model residuals (ESM 5). Model predictions highlight the coastal waters to the south of the South Shetland Islands within the Bransfield Strait, and along the shelf break to the north of the South Shetland Islands as areas with elevated krill density (Fig. 4), which coincides with areas where increased krill density was observed. Lowest winter krill density was predicted to the north of the South Shetland Islands, both on- and off-shelf, and towards the tip of the Antarctic Peninsula. Spatial error was highest in areas with the highest predicted krill density (Fig. 4).

## Discussion

Our model aggregates integrated krill density derived from acoustic data collected over 11 summers, and 4 winters to create seasonal average distribution and density estimates of krill. The maximum observed krill density per grid cell in different years ranged from 179 to 2272 g m^−2^ (summer) and 64 to 1880 g m^−2^ (winter), highlighting the extreme patchiness of krill density in this region. Importantly, our modelling approach does not capture inter-annual variation in krill biomass, rather it focuses on identifying areas of predictable krill occurrence and capturing average conditions, both important management issues.

Our habitat models build on previous studies about krill distribution in this area (e.g. Santora et al. 2012; Silk et al. 2016; Reiss et al. 2017), associating krill distribution with environmental characteristics, often with similar results. However, in this study, the models are also used to predict spatial layers which describe the seasonal distribution of krill at a fine-scale. By visualising the predicted distributions in this way, we are better able to highlight the areas where increased krill density is expected. Additionally, by integrating predicted krill density with information about the distribution and krill requirements of krill-dependent predators (e.g. Warwick-Evans et al. in press), CCAMLR should be able to develop an evidence-based approach to the management of the krill fishery, incorporating the ecology of predators and krill at relevant spatial scales.

### Model performance and complexity

Areas where our models predicted high krill density correspond with observed areas of elevated krill density that are consistent across years. For example, in many years, elevated summer krill density was observed in the waters surrounding Elephant Island, and in the nearshore areas immediately to the north of the Peninsula as well as to the north of the South Shetland Islands. These areas coincide with the shelf break along the north of the South Shetland Islands and to the north of Elephant Island, and a series of canyons within the Bransfield Strait and to the north of the Antarctic Peninsula. Predictions from our model highlight these same areas of increased krill density, suggesting that our model performs well in identifying areas where krill density is consistently elevated. It is possible that these similarities are a result of overfitting the model to the dataset, however, the models were constrained to reduce overfitting, and response curves show similar patterns to previous findings. For example, increased krill density associated with the shelf break and with moderate concentrations of chlorophyll-a (Atkinson et al. 2008; Silk et al. 2016). As such we believe that these similarities are not a result of model overfitting. Similarly, during winter, elevated krill density was observed in the Bransfield Strait, and in nearshore waters surrounding the South Shetland Islands, areas also highlighted by our model predictions, and consistent with previous findings (Lascara et al. 1999; Reiss et al. 2017; Siegel 1988). However, our model predictions also suggest areas between the South Shetland Islands and Elephant Island have elevated krill density during winter, which is not consistent with sampling observations. As such, we recognise that predictions from the winter model may be less robust than those for summer. This may be because our models have not fully captured the drivers of krill distribution, particularly if krill actively migrate, in which case there may be areas of suitable habitat that krill are choosing not to use. Additionally, fewer data have been collected during winter, reducing the scope for capturing long-term patterns.

Our model predictions are also consistent with the local ecology. For example, penguin colonies are only likely to establish in areas where prey availability is predictable between years and where biomass is sufficient to sustain the size of the colony (Ichii et al. 1996; Trivelpiece and Fraser 1996). Large penguin colonies exist on Elephant Island, Lowe Island and on the surrounding islets (Humphries et al. 2017), coinciding with the predicted elevated krill densities in our models in these areas during summer. Additionally, our model predictions are consistent with the recent distribution of the commercial krill fishery, which prioritises fishing in predictable locations with high krill biomass, having become more concentrated in the nearshore waters of the northern Antarctic Peninsula in recent years (Trathan et al. 2018).

Acoustic methods to estimate biomass density from acoustic trawl surveys have several sources of uncertainty associated with them (Demer et al. 2004), including those associated with the target strength model, errors in the length frequency distributions of krill and the angle of krill in the water column relative to the acoustic beams. Therefore, inter-annual variability (order of magnitude) and survey uncertainty in acoustic estimates, which can be large (CVs > 30%), may have added uncertainty into the spatial habitat model developed here. However, because the spatial distribution of biomass density used is the average over a number of summer or winter surveys, the average distribution used in the habitat model may be robust to these sources of variability and uncertainty. Further, it is likely that uncertainty introduced in model processing is consistent within the dataset, and that areas where increased krill density was observed are indeed areas of higher krill density, although the density estimate may be slightly inaccurate.

### Krill habitat characteristics

Krill life-history, in the context of its biological and physical environment, dictates its distribution and abundance. During summer, krill are distributed both in shelf waters and in adjacent deep-water habitats with ~ 90% of krill biomass occurring off-shelf (Atkinson et al. 2008). Spawning occurs during austral summer, both on- and off-shelf (Perry et al. 2019), although Siegel et al. 2013 and Hofmann and Hüsrevoğlu 2003 suggest spawning is more successful along the shelf break and in oceanic waters than in shelf waters. During autumn and winter, a shelf-ward migration occurs (Siegel 1988), and estimates of krill biomass in the Bransfield Strait during winter are more than an order of magnitude higher than in summer (Siegel 1988; Reiss et al. 2017). However, krill may also overwinter in deep-ocean habitats (Lascara et al. 1999; Siegel 2005). Within these broad patterns of krill distribution associated with life-history processes, additional habitat features may be associated with areas of increased krill distribution.

Previous studies attempting to identify the drivers behind the distribution of krill have failed to identify a unifying driver of krill distribution (e.g. Trathan et al. 2003; Silk et al. 2016). As such, the habitat descriptors included in our models may not necessarily be the overall drivers of krill distribution. This highlights the challenges associated with modelling the distribution of krill, especially given the high inter-annual variation in krill density. However, our model predictions indicate predictable areas of increased krill density across the study area and our analysis provides an important step forward for modelling krill distribution. We explore possible reasons for such associations below.

#### Summer

Our model predicted higher krill density near the shelf break, in warmer more saline water with medium–high chlorophyll-a concentration. Elevated summer krill density has frequently been observed along the shelf break and on-shelf waters (e.g. Trathan et al. 2003; Klevjer et al. 2010; Silk et al. 2016). Although up to 90% of krill abundance is estimated to occur in off-shelf waters during summer (Atkinson et al. 2008), krill density is frequently higher in coastal and shelf waters during this time (Trathan et al. 2003; Siegel 2005; Warren and Demer 2010; Silk et al. 2016). This is most likely due to interactions between behaviour, advection by local currents and retention of krill in shelf waters (Young et al. 2014), potentially combined with an influx of nutrient-rich water increasing phytoplankton biomass (Prézelin et al. 2000) and providing a food supply for krill. Additionally, krill may aggregate over the shelf to avoid predation, which may be less important off-shelf, especially if krill predators occur at lower densities (Reid et al. 2004); certainly models that include active krill behaviour result in distribution patterns that are associated with increased survival (Richerson et al. 2015) growth, and reproductive success.

Along the western Antarctic Peninsula, krill are transported by ocean currents along the shelf break (Ichii et al. 1998; Siegel 2005; Piñones et al. 2013). Canyons and other coastal topographical features may therefore provide refugia from currents that would otherwise advect krill away from the region (Lawson et al. 2008). The topography of the southern Bransfield Strait is highly complex with many submarine canyons, which may enable krill to aggregate within topographical features, despite the strong current flow.

Chlorophyll-a concentration is frequently used as a proxy for food availability. It is hypothesised that increased krill density at moderate chlorophyll-a concentrations results from a trade-off between increased food availability and predation risk (Atkinson et al. 2008). We estimate a peak in krill density at moderate concentrations of chlorophyll-a (~ 2 mg m^3^) which is consistent with previous findings (e.g. Atkinson et al. 2008; Silk et al. 2016). Silk et al. (2016) reported that krill densities tended to be higher at chlorophyll-a concentrations of 0.3–1.4 mg m^−3^ in the west Antarctic Peninsula region. However, Silk et al. (2016) could not find consistent relationships across the wider Scotia Sea, suggesting relationships with chlorophyll-a are not consistently observed (e.g. Santora et al. 2012; Siegel et al. 2013).

Variability in salinity and sea surface temperature may be a result of influx of different water masses from different parts of the Southern Ocean (Fig. 2; ; Sangrà et al. 2011; Moffat and Meredith 2018; Trathan et al. 2018), Winter Water formation, or localised melting of glacial ice (Cook et al. 2016). In the Bransfield Strait, Sangrà et al. 2011 suggest two water masses are important—Bellingshausen-influenced and Weddell Sea-influenced waters which lead to a system of anticyclonic eddies in the central Bransfield Strait (Sangrà et al. 2011); such eddies could be important in the transport or retention of krill (Reiss et al. 2020.

Waters from the Bellingshausen Sea are characterised by a surface layer of Antarctic Surface Water (AASW) over a deep layer of Circumpolar Deep Water (CDW, e.g. Moffat et al. 2009). CDW comprises the Upper CDW, which has a temperature maximum of 1.55 to 2.10 °C and salinities of 34.62 to 34.68. (Sievers and Nowlin Jr. 1984), and Lower CDW, which is colder (1.25 to 1.57 °C) and saltier (salinities ~ 34.73) (Sievers and Nowlin 1984). At the Antarctic Peninsula, the UCDW intrudes over the shelf, whilst LCDW is found in several deep canyons and depressions connected to the shelf break.

The Weddell Gyre has a cold, low salinity surface overlying a thick relatively warm (~ 0.50 °C) and salty (~ 34.69) layer (Muench and Gordon 1995). The outflow of the Weddell Sea influenced water floods the southern Bransfield Strait shelf area. This region is now of key importance to the krill fishery. The US AMLR acoustic surveys have little spatial coverage in this area (Fig. 1), having been chosen prior to the fishery moving to that area. The influence of saline waters in our models suggests the Weddell Sea is plausibly an important source of krill in the area, consistent with findings by (Siegel et al. 2013).

In the Bransfield Strait, the basic circulation patterns consist of a western inflow of relatively warm water from the Bellingshausen Sea, the Gerlache Strait and the Circumpolar Current, and an eastern inflow of relatively cold water from the Weddell Sea (Fig. 2; Sangrà et al. 2011).

Our models highlight the positive relationship between krill abundance and salinity values expected in Weddell-influenced and Bellingshausen-influenced waters. However, the relative influence of these two water masses as sources of krill remains an active topic of investigation (Trathan et al. 2021). Variability in the absolute, and relative, contributions of krill through the different oceanographic gateways into the Bransfield Strait will be of key importance for management (Trathan et al. 2021).

#### Winter

The best model to describe winter krill distribution predicted increased krill density in shallow waters, peaking at ~ 1500 m, with low sea-ice concentration, medium sea level anomaly and moderate or high water velocity, peaking at ~ 0.2 m s^−1^, and increasing again at values over 0.6 m/s. Increased krill density in shallow waters (< 1500 m) during winter supports the theory that krill undergo seasonal migration onto the shelf, and is consistent with previous findings (e.g. Nicol 2006; Reiss et al. 2017; Siegel 1988). A common hypothesis suggests that krill migrate on-shelf to feed on ice-algae under the sea-ice as phytoplankton levels in the water column decrease in winter (Marschall 1988; Ryabov et al. 2017), and evidence suggests that both larval and adult krill are closely associated with sea-ice (Kawaguchi and Satake 1994; Loeb et al. 1997; Daly 2004). However, recent studies which support the on-shelf migration hypothesis suggest migration is independent of sea-ice conditions, and that though sea-ice provides shelter it is a food-poor habitat (Meyer et al. 2017; Reiss et al. 2017; Walsh et al. 2020). Our models show a negative relationship between krill density and sea-ice, and previous studies using Remotely Operated Vehicles or divers have also found no evidence of adult krill under the sea-ice during winter (Quetin et al. 1996; Lawson et al. 2008), but see also Brierley et al. 2002. An alternate hypothesis, that during winter krill may migrate to deeper water (beyond the 250 m limit of many scientific surveys) to feed, has been recognised (Lascara et al. 1999; Siegel 2005), but does not explain the on-shelf migration behaviour. On-shelf movement must be a result of active migration as the currents in the region would not aggregate krill on-shelf during this time (Reiss et al. 2017), thus more emphasis on the potential characteristics that could provide organisational cues for krill aggregating over winter is required.

Our models suggest that areas of elevated krill density may be associated with moderate water velocity and sea level anomaly. Coastal currents within the Bransfield Strait, or eddies and fronts, indicated by sea level anomaly may aggregate krill in these environments (Santora et al. 2012). Acoustic surveys only represent a brief snapshot of a very dynamic ecosystem, comprising a complex mosaic of habitats. Process studies will therefore be needed to improve our understanding about krill behaviour in relation to habitat characteristics.

#### Seasonal comparison

Our models support the seasonal krill migration hypothesis, as the predicted distribution of krill becomes more concentrated in the Bransfield Strait during winter. It would be interesting to understand the direction in which the higher densities of krill observed around Elephant Island move during winter, whether into the coastal areas of Elephant Island, or towards the South Shetland Islands, however, data are not yet available with which to fully understand this situation. Given the complexity of the shelf break in the Antarctic Peninsula, ontogenetic migrations are likely to be complex and possibly differ within the region.

During summer, our models predicted the presence of krill throughout the region, with generally, a higher overall mean density, and with higher density patches than in winter. During winter, our models indicated krill were absent throughout much of the region, with generally, a lower mean density than in summer. Importantly, our models predict the average distribution of krill, with spatio-temporal smoothing. As such, the size and depth of individual krill patches, layers or swarms are not evident from our model predictions. However, understanding the variability in the size, depth and extent of krill swarms will be important as management of the krill fishery develops, given that the primary focus of industry is to target concentrated krill (Santa Cruz et al. 2018; Trathan and Hill 2016). Therefore, in future, it would be interesting to look at seasonal variation in the distribution of krill, particularly in relation to the distribution and density of swarms, given that krill are highly dynamic and occur in both loose layers and dense swarms (Miller and Hampton 1989). Indeed, Lascara et al. (1999) have reported that during summer krill are concentrated in the upper 50 m of the water column, whilst in winter they generally occur at depths > 100 m. Lascara et al. 1999 also noted that during winter, high-density swarms were considerably larger than during summer (~ 10 km and 2 km respectively), consistent with Reiss et al. (2017). In future studies, it would therefore be interesting to look at seasonal variation in the depth and spatial distribution of fishable aggregations of krill.

Additionally, it is likely that krill distribution varies according to life-stage. For example, in the Antarctic Peninsula and South Shetland Islands region, larger krill are found mainly in the open ocean and along the shelf break during spring and summer and juvenile krill occupy the inner shelf waters (Atkinson et al. 2008; Reiss et al. 2008; Siegel et al. 2013). Acoustic sampling increases information about the distribution of juvenile and adult krill (but not early life-stages), although it does not allow us to differentiate between juveniles, adults or spawning females. As such, it is not possible to describe the distribution of krill in our models according to demographics. However, it may be beneficial to protect some demographic classes of krill over others (e.g. spawning females), and as such, demographic variability in distribution should be considered in the future for fisheries management.

### Implications for fisheries management

Our krill habitat models provide vital information for use in the current ecosystem approach to fisheries management endorsed by CCAMLR. By understanding where krill density may be elevated, and where predators depend upon these resources, we can identify the key areas where harvesting krill will cause minimum impact.

The U.S. AMLR survey was designed to evaluate ecosystem variability in the northern Antarctic Peninsula region, including in the area historically used by the krill fishery. However, over time, the fishery has changed in both location and timing (CCAMLR 2018). Consequently, the operational area now used by the fishery extends beyond the area covered by the U.S. AMLR survey (Reiss et al. 2008, 2017), with fishing effort focussed in nearshore waters (Fig. 2; Trathan et al. 2018). Furthermore, the fishery preferentially operates in the autumn and early winter period (March, April and May; see Trathan et al. 2021), whereas the U.S. AMLR survey occurred between January and early March (Reiss et al. 2008), and during August and September (Reiss et al. 2017). The fishery presumably focusses operations in nearshore areas during autumn because these areas contain the most predictable and profitable krill aggregations (Trathan et al. 2021).

The recent spatio-temporal shift in the krill fishery is plausibly, at least partially, a reflection of a change in the underlying accessibility in the distribution of krill (Silk et al. 2014). Concurrently, trends in the distribution and abundance of krill-dependent predators in the region have been observed. Cetaceans, fur seals and finfish are recovering after being harvested to near extinction (Hucke-Gaete et al. 2004; Branch 2011; Barrera-Oro et al. 2017), with humpback whales estimated to have recovered (Jackson et al. 2015), elsewhere in the western South Atlantic to 93% of pre-harvesting levels (Zerbini et al. 2019). As previously depleted krill-dependent predator populations recover, competition between predator species is likely to occur, possibly resulting in a change to ecosystem dynamics. Indeed, chinstrap penguin populations in the region have declined (Strycker et al. 2020). Moreover, there remains conflicting evidence as to whether krill biomass is declining, possibly as a consequence of climate change (Atkinson et al. 2019; Cox et al. 2018), although impacts of changing climate on the ecosystem in this area have already been observed (Loeb et al. 1997; Moline et al. 2004; Mendes et al. 2018). In light of these changes to the ecosystem, we caution about whether the distribution and density of krill predicted by our models remain current. For example, the data parameterised in our summer models are 10 to 20 years old, and those for winter, although more recent, are still outdated. Indeed, it is plausible that the differences in the predicted distribution of krill between summer and winter may be partially a result of the time-frame in which the surveys were undertaken. However, our findings are comparable with previous studies, and as such are likely to be reasonable. Certainly, the data we use are the most up-to-date available.

Alongside the ecosystem changes in the Peninsula region, the krill fishery has also evolved. A regime shift from midwater trawl fisheries to continuous fishing, where the catch is pumped directly from the cod-end to the ship, increases the efficiency of the fishery. Simultaneously, catches have increased to their highest values since the 1980s (CCAMLR 2020). As such, it is plausible that models based on data from decades past may not completely encapsulate these changes. We highlight the necessity that management of the krill fishery remains precautionary, up-to-date krill surveys at relevant spatial and temporal scales are urgently required.

Our models suggest that during summer, krill density is elevated to the north of the South Shetland Islands, whereas during winter elevated densities are predicted in the Bransfield Strait. Maximum krill density across the study area is predicted to be higher in summer than in winter. This highlights concerns about using density data collected during summer within the management framework for the krill fishery which operates mostly between March and May (Trathan et al. In press). It also highlights the need to further understand links between the distribution of krill during summer and winter. It is increasingly recognized that miss-matches in the scale of management and important ecological process are likely to undermine management actions. Collection of data on krill distributions for management of the krill fishery at relevant scales will benefit from a diverse portfolio of sampling methods, which will include research vessels, the fishery (Watkins et al. 2016), and other platforms such as gliders (Guihen et al. 2014; Reiss et al. 2021) and moorings (Brierley et al. 2006).

We emphasise that currently the U.S. AMLR survey remains the best available source of acoustic information for krill in the northern Peninsula region. However, given that the area in which the fishery operates extends further south and into the Gerlache Strait (Trathan et al. 2018), it is likely that any management strategy which encompasses the entire area used by the fishery will need to rely upon extrapolation into un-surveyed areas. Models based on the U.S. AMLR survey data will be the most reliable until further data are available, including in near shore areas and at the most appropriate time of year. Observations to validate our model predictions especially outside of the current study area will almost certainly require new survey effort in both space and time. There is the potential that fishing vessels could be used to sample krill density, as they do in the South Orkney Island (Krafft 2015; Kraft et al. 2021). Indeed, this approach is being discussed within CCAMLR for implementation in the Antarctic Peninsula region.

We highlight that any future krill management strategy must be robust to different aspects of uncertainty. The role of ocean currents in krill distribution and movement has been a topic of intense debate over a number of decades (e.g. Hofmann et al. 1998; Nicol 2006). Whether or not krill exist as an isolated and self-sustaining localised stock, or as part of a larger [Scotia Sea or circumpolar] stock that moves with ocean currents, or as a combination of both small and larger-scale components remains to be fully determined. Additionally, an understanding of the potential changes that may occur as the climate continues to alter should be considered in order to ensure that management frameworks are robust to future modifications. These may result in particular spatial bottlenecks, for example a possible lack of suitable habitat (for spawning, recruitment and overwintering), or increased interactions with the fishery as reduced sea-ice enables the fishery to operate in previously unused areas (Nicol et al. 2012).

## Conclusion

The predictions from our krill model represent the temporal average pattern of krill distribution, recognising that inter-annual variability also occurs. We believe that our models can be used to provide projections into local areas adjacent to the U.S. AMLR study area and covering the same extent as current fishery operations in the region. However, it is vital that these model predictions are validated with new observations, including in areas without data but in which the fishery operates, particularly in winter and in regions that are not currently surveyed by vessels with acoustic capability. As such, model predictions covering areas used by the fishery (and by predators) remain fundamental to the development of a management strategy recently endorsed by CCAMLR.

## Supplementary Information

Below is the link to the electronic supplementary material.

Supplementary file1 (PDF 160 KB)

Supplementary file2 (PDF 186 KB)

Supplementary file3 (PDF 111 KB)

Supplementary file4 (PDF 125 KB)

Supplementary file5 (PDF 75 KB)
