# Supplementary material for: Estimating the average distribution of Antarctic krill Euphausia superba at the northern Antarctic Peninsula during austral summer and winter
Source: Polar Biol. 2022 Apr 15;45(5):857–71. doi: 10.1007/s00300-022-03039-y (PMC9165435; doi:10.1007/s00300-022-03039-y)
Supplement: Supplementary file 1 — Supplementary file1 (PDF 160 KB) [file 300_2022_3039_MOESM1_ESM.pdf]

Electronic Supplementary Materials 1

Model selection by cross validation

Model performance was evaluated using k-fold cross validation: the data were split into 6 groups, and models were developed using data from all but one group. The model was then used to predict the krill density for the excluded group and was evaluated using the normalised root mean square error (NMRSE). Lower values of NMRSE indicate a better model fit. This process was carried out excluding each group in turn to ensure that all years were used for model evaluation, and the mean NMRSE across years was taken.

Table S1 NRMSE evaluations for GAMS modelling the density of Antarctic krill *Euphausia superba* during summer, using 6 fold cross validation.

Covariate	NRMSE
**S(Salinity)**	**0.0852**
S(Chlorophyll)	0.0857
S(Distance to shelf break)	0.0859
S(Sea Surface Temperature)	0.0862
S(Depth)	0.0863
S(Mean Sea Level Anomaly)	0.0868
S(Slope)	0.0870
S(Current speed)	0.0872
S(Eddy kinetic energy)	0.0875
S(Chlorophyll lag 1)	0.0879
S(Chlorophyll lag 2)	0.0879
Null	0.0880
**S(Salinity) + S(Chlorophyll)**	**0.0841**
S(Salinity) + S(Sea Surface Temperature)	0.0844
S(Salinity) + S(Distance to shelf)	0.0845
S(Salinity) + S(Slope)	0.0847
S(Salinity) + S(Depth)	0.0847
S(Salinity) + S(Current speed)	0.0849
S(Salinity) + S(Eddy Kinetic Energy)	0.0850
S(Salinity) + S(Mean Sea Level Anomaly)	0.0852
**S(Salinity) + S(Chlorophyll) + S(Distance to shelf)**	**0.0830**
S(Salinity) + S(Chlorophyll) + S(Sea Surface Temperature)	0.0833
S(Salinity) + S(Chlorophyll) + S(Slope)	0.0836
S(Salinity) + S(Chlorophyll) + S(Depth)	0.0836
S(Salinity) + S(Chlorophyll) + S(Water Velocity)	0.0838
S(Salinity) + S(Chlorophyll) + S(Eddy Kinetic Energy)	0.0840
S(Salinity) + S(Chlorophyll) + S(Mean Sea Level Anomaly)	0.0841
**S(Salinity) + S(Chlorophyll) + S(Distance to shelf) + S(Sea Surface Temperature)**	**0.0819**
S(Salinity) + S(Chlorophyll) + S(Distance to shelf + S(Slope)	0.0825
S(Salinity) + S(Chlorophyll) + S(Distance to shelf + S(Depth)	0.0824
S(Salinity) + S(Chlorophyll) + S(Distance to shelf + S(Water Velocity)	0.0827
S(Salinity) + S(Chlorophyll) + S(Distance to shelf + S(Eddy Kinetic Energy)	0.0829
S(Salinity) + S(Chlorophyll) + S(Distance to shelf + S(Mean Sea Level Anomaly)	0.0830

Table S2 NRMSE evaluations for GAMS modelling the density of Antarctic krill *Euphausia superba* during winter, using 6 fold cross validation.

Covariate	NRMSE
**S(Depth, k=5)**	**0.0851**
S(Mean Sea Level Anomaly, k=6)	0.0859
S(Salinity, k=5)	0.0861
S(Sea Surface Temperature, k=4)	0.0868
S(Distance to shelf break, k=7)	0.0869
S(Sea-ice concentration, k=4)	0.0870
S(Sea-ice concentration lag 2 week, k=4)	0.0871
S(Sea-ice concentration, lag 1 month k=4)	0.0873
S(Sea-ice concentration, lag 2 months k=4)	0.0871
S(Chlorophyll, k=5)	0.0874
S(Current speed, k=4)	0.0877
S(Slope, k=4)	0.0878
S(Eddy kinetic energy, k=4)	0.0879
Null	0.0878
**S(Depth, k=5) + S(Mean Sea Level Anomaly, k=6)**	**0.0849**
S(Depth, k=5) + S(Sea-ice concentration, k=4)	0.0851
S(Depth, k=5) + S(Sea Surface Temperature, k=4)	0.0856
S(Depth, k=5) + S(Salinity, k=5)	0.0860
S(Depth, k=5) + S(Chlorophyll, k=5)	0.0865
S(Depth, k=5) + S(Slope, k=4)	0.0867
S(Depth, k=5) + S(Current speed, k=4)	0.0873
S(Depth, k=5) + S(Eddy kinetic energy, k=4)	0.0874
**S(Depth, k=5) + S(Mean Sea Level Anomaly, k=6) + S(Sea-ice concentration, k=4)**	**0.0841**
S(Depth, k=5) + S(Mean Sea Level Anomaly, k=6) + S(Current speed, k=4)	0.0846
S(Depth, k=5) + S(Mean Sea Level Anomaly, k=6) + S(Sea Surface Temperature, k=4)	0.0847
S(Depth, k=5) + S(Mean Sea Level Anomaly, k=6) + S(Salinity, k=5)	0.0848
S(Depth, k=5) + S(Mean Sea Level Anomaly, k=6) + S(Chlorophyll, k=5)	0.0849
S(Depth, k=5) + S(Mean Sea Level Anomaly, k=6) + S(Slope, k=4)	0.0850
S(Depth, k=5) + S(Mean Sea Level Anomaly, k=6) + S(Eddy kinetic energy, k=4)	0.0850
**S(Depth, k=5) + S(Mean Sea Level Anomaly, k=6) + S(Sea-ice concentration, k=4) + S(Chlorophyll, k=5)**	**0.0835**
S(Depth, k=5) + S(Mean Sea Level Anomaly, k=6) + S(Sea-ice concentration, k=4) + S(Current speed, k=4)	0.0836
S(Depth, k=5) + S(Mean Sea Level Anomaly, k=6) + S(Sea-ice concentration, k=4) + S(Sea Surface Temperature, k=4)	0.0839
S(Depth, k=5) + S(Mean Sea Level Anomaly, k=6) + S(Sea-ice concentration, k=4) + S(Salinity, k=5)	0.0839
