# Supplementary material for: Estimating the average distribution of Antarctic krill Euphausia superba at the northern Antarctic Peninsula during austral summer and winter
Source: Polar Biol. 2022 Apr 15;45(5):857–71. doi: 10.1007/s00300-022-03039-y (PMC9165435; doi:10.1007/s00300-022-03039-y)
Supplement: Supplementary file 2 — Supplementary file2 (PDF 186 KB) [file 300_2022_3039_MOESM2_ESM.pdf]

Electronic Supplementary Material 2

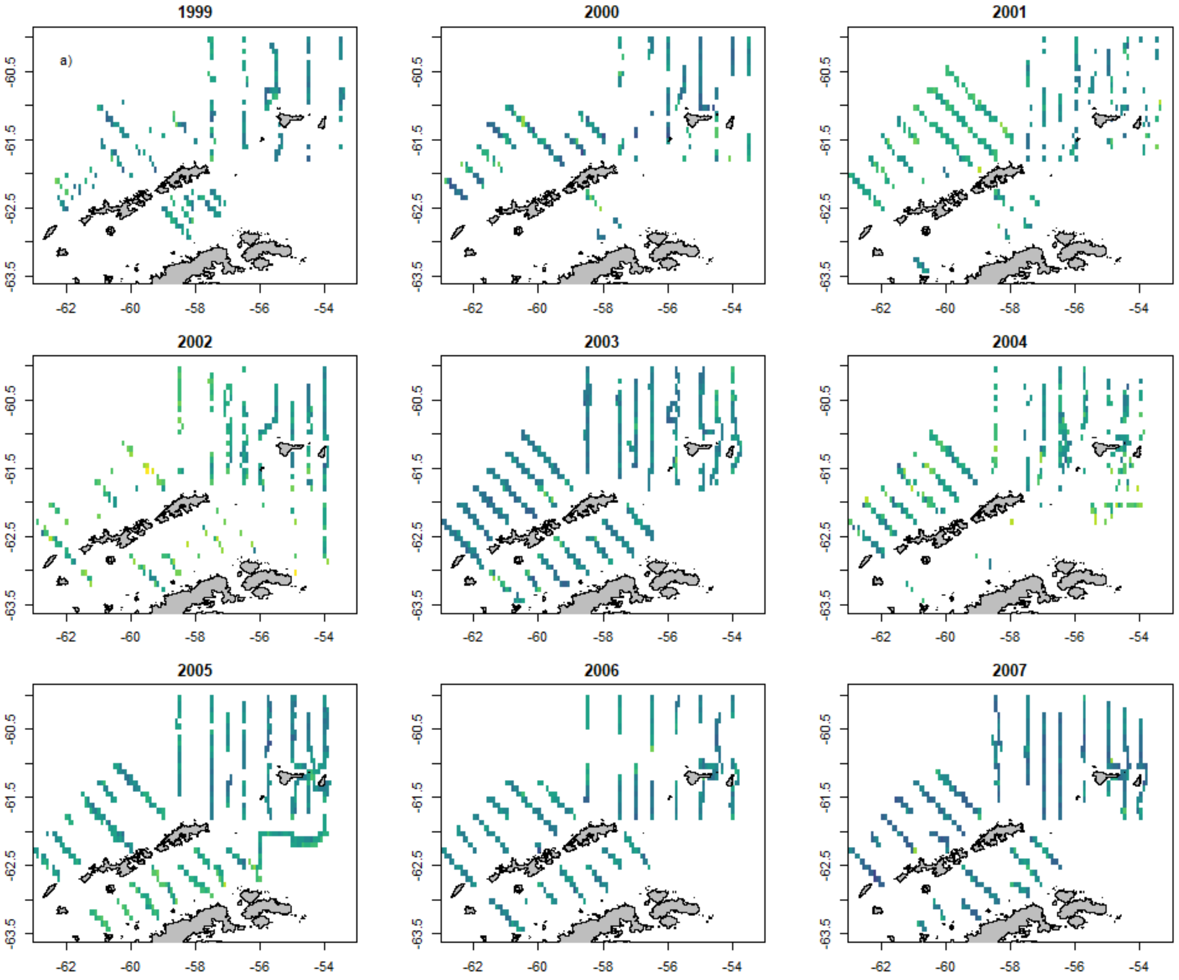

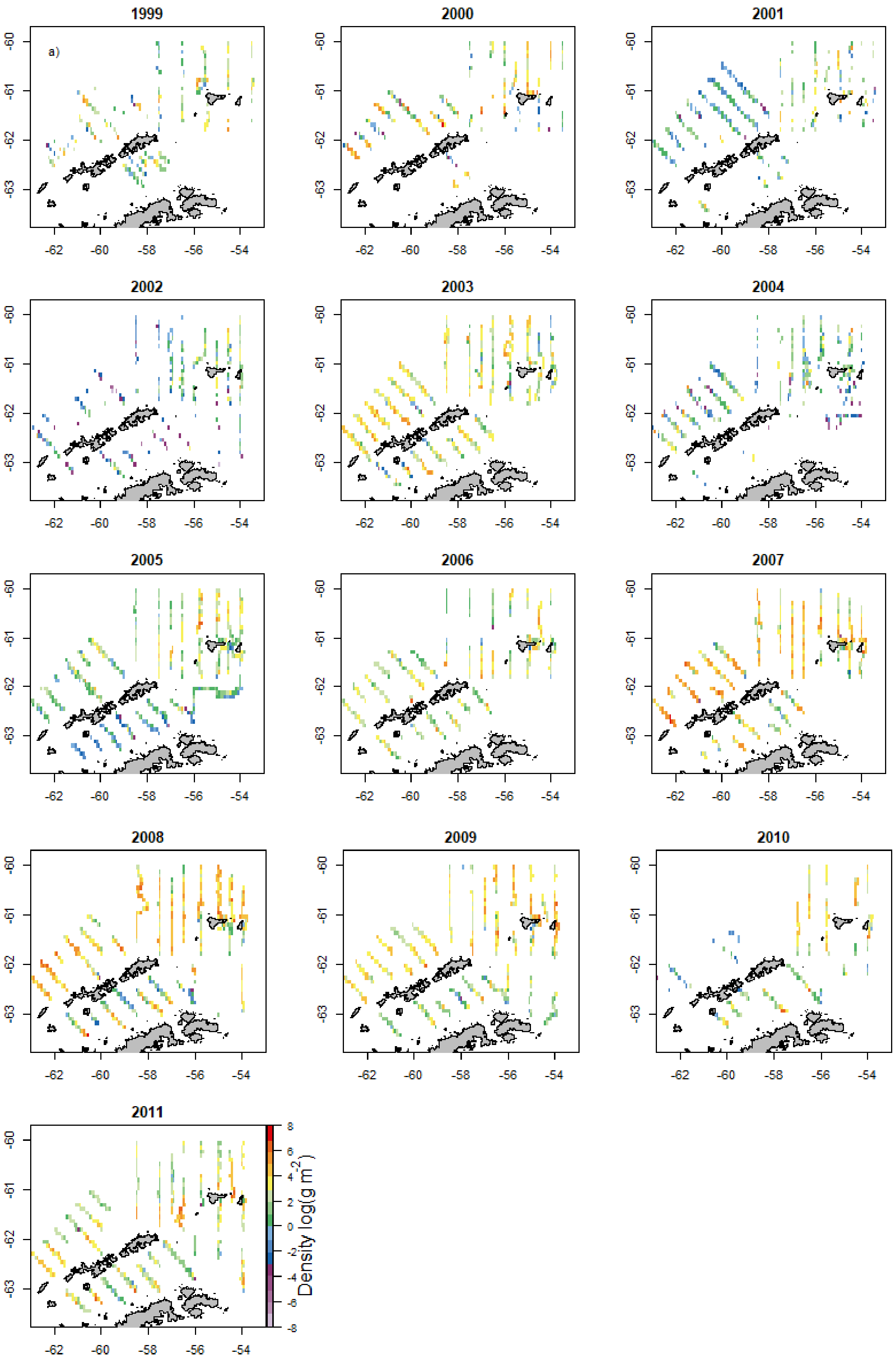
1999
2000
2001
2002
2003
2004
2005
2006
2007
2008
2009
2010
2011
a)
-60
-61
-62
-63
-62
-60
-58
-56
-54
8
6
4
2
0
-2
-4
-6
-8
Density log(g m^-2)

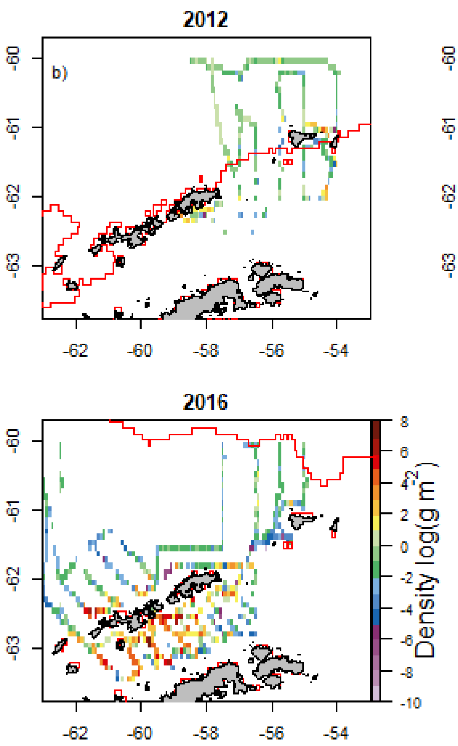

Figure S1. Mean density (log, gm^{-2}) of Antarctic krill *Euphausia superba* for all survey years during a) summer (1999 to 2011), b) winter (2012-2016). The mean extent of winter (August-September) sea-ice concentration >15% is shown in red on the winter plots. During 2015 the sea ice extent (>15%) was further north than our study area.
