# Supplementary material for: Estimating the average distribution of Antarctic krill Euphausia superba at the northern Antarctic Peninsula during austral summer and winter
Source: Polar Biol. 2022 Apr 15;45(5):857–71. doi: 10.1007/s00300-022-03039-y (PMC9165435; doi:10.1007/s00300-022-03039-y)
Supplement: Supplementary file 3 — Supplementary file3 (PDF 111 KB) [file 300_2022_3039_MOESM3_ESM.pdf]

Electronic Supplementary Material 3

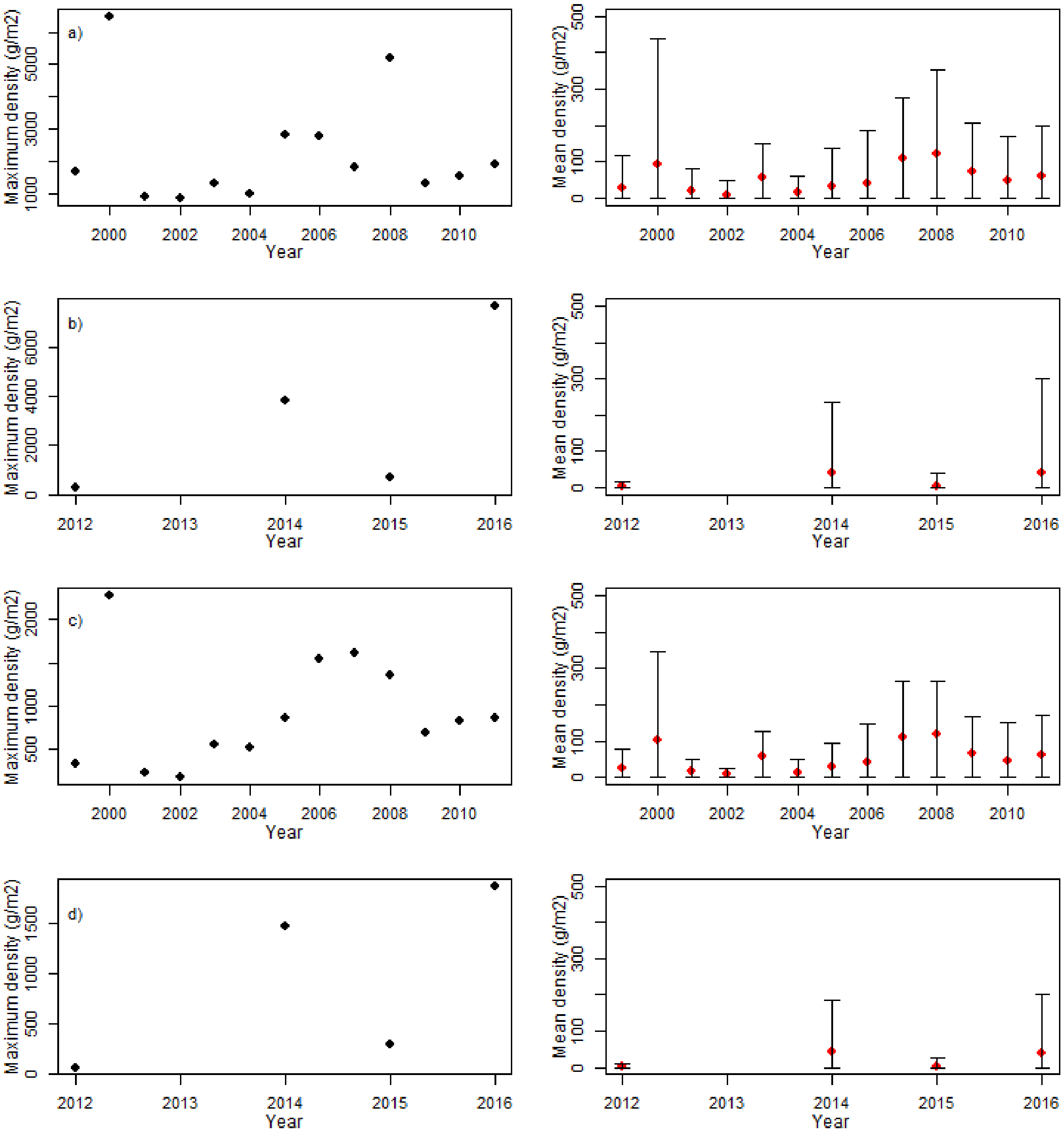

Figure S2. The annual maximum and mean (± sd) density of Antarctic krill *Euphausia superba* a) individual raw samples during summer b) individual raw samples during winter c) averaged within grid cells during summer d) averaged within grid cells during winter. Maximum krill density is lower in years where the extent of the survey area was more limited (see Figure S1). As such, these values may be an artefact of survey coverage.
