# Supplementary material for: Estimating the average distribution of Antarctic krill Euphausia superba at the northern Antarctic Peninsula during austral summer and winter
Source: Polar Biol. 2022 Apr 15;45(5):857–71. doi: 10.1007/s00300-022-03039-y (PMC9165435; doi:10.1007/s00300-022-03039-y)
Supplement: Supplementary file 4 — Supplementary file4 (PDF 125 KB) [file 300_2022_3039_MOESM4_ESM.pdf]

Electronic Supplementary Materials 4

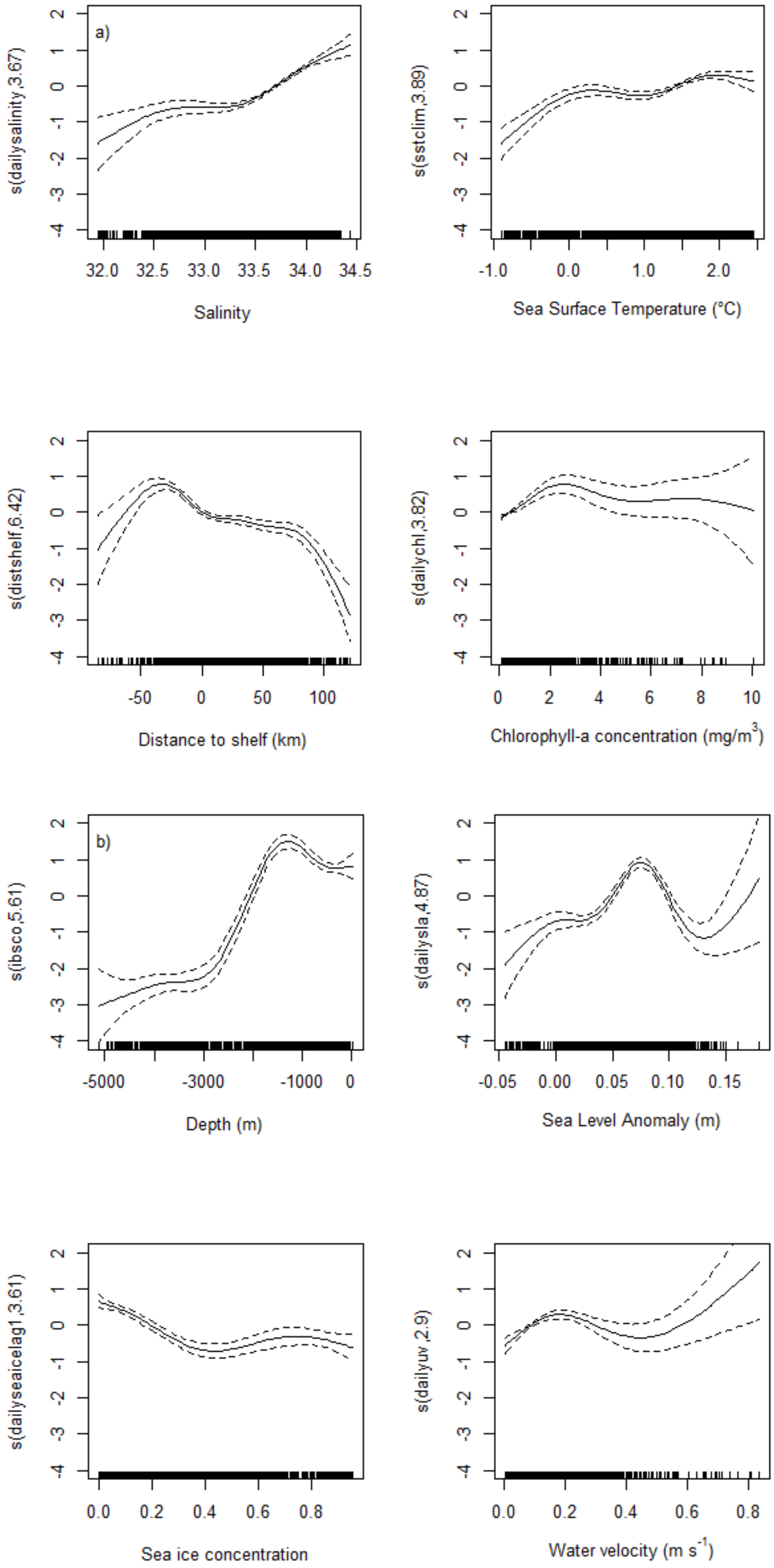

Figure S3. Response curves (smooth curve ± standard error) from models to plot the distribution and density of Antarctic krill *Euphausia superba* in the Antarctic Peninsula region during a) summer, b) winter. Vertical lines along the bottom represent the empirical data values). Winter values for sea

level anomaly and water velocity in the grid into which we were predicting did not exceed 0.15 and 0.5, respectively, thus the values where these response curves show much higher uncertainty were not used. If the model were to be used to predict the krill distribution in areas where these higher values of sea level anomaly and water velocity were present, they should be interpreted with caution as the response to these covariates is much more uncertain at these higher values.
