# Supplementary material for: Estimating the average distribution of Antarctic krill Euphausia superba at the northern Antarctic Peninsula during austral summer and winter
Source: Polar Biol. 2022 Apr 15;45(5):857–71. doi: 10.1007/s00300-022-03039-y (PMC9165435; doi:10.1007/s00300-022-03039-y)
Supplement: Supplementary file 5 — Supplementary file5 (PDF 75 KB) [file 300_2022_3039_MOESM5_ESM.pdf]

Electronic Supplementary Material 5

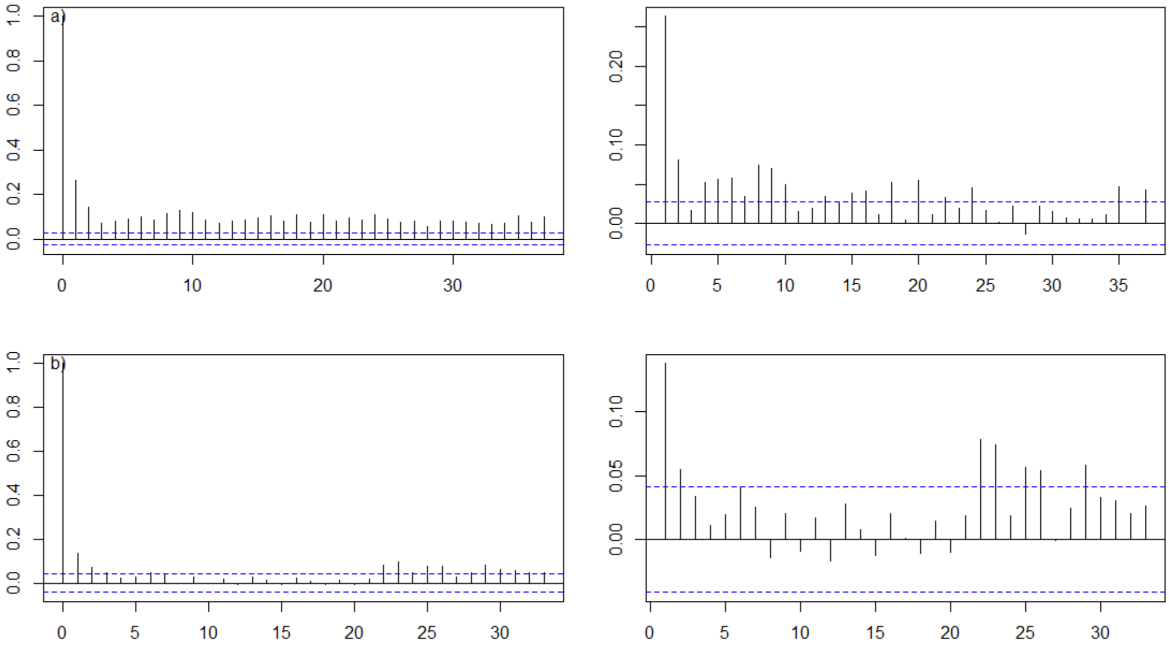

Figure S4 Autocorrelation function (ACF) and Partial (ACF) plots for model residuals during a) summer, b) winter. Where the ACF values are above the blue line, the autocorrelation is significant. However, the low values of ACF, and the lack of a decreasing trend or pattern in the ACF values suggest that this residual autocorrelation is minimal.
